# A Novel Competitive Binding Screening Assay Reveals Sennoside B as a Potent Natural Product Inhibitor of TNF-α

**DOI:** 10.3390/biomedicines9091250

**Published:** 2021-09-17

**Authors:** Lei Peng, Prasannavenkatesh Durai, Keunwan Park, Jeong Joo Pyo, Yongsoo Choi

**Affiliations:** 1 School of Chemistry and Chemical Engineering, Qiqihar University, Qiqihar 161006, China; 03555@qqhru.edu.cn; 2 Natural Product Informatics Research Center, Korea Institute of Science and Technology (KIST), Gangneung 25451, Korea; prasanna@kist.re.kr (P.D.); keunwan@kist.re.kr (K.P.); 3 Natural Product Research Center, Korea Institute of Science and Technology (KIST), Gangneung 25451, Korea; 4 Department of Biological Chemistry, University of Science and Technology, Daejeon 34113, Korea

**Keywords:** tumor necrosis factor α, natural products, sennoside B, analytical size exclusion chromatography, liquid chromatography-tandem mass spectrometry

## Abstract

Natural products (NPs) have played a significant role in drug discovery for diverse diseases, and numerous attempts have been made to discover promising NP inhibitors of tumor necrosis factor α (TNF-α), a major therapeutic target in autoimmune diseases. However, NP inhibitors of TNF-α, which have the potential to be developed as new drugs, have not been reported for over a decade. To facilitate the search for new promising inhibitors of TNF-α, we developed an efficient competitive binding screening assay based on analytical size exclusion chromatography coupled with liquid chromatography-tandem mass spectrometry. Application of this screening method to the NP library led to the discovery of a potent inhibitor of TNF-α, sennoside B, with an IC_50_ value of 0.32 µM in TNF-α induced HeLa cell toxicity assays. Surprisingly, the potency of sennoside B was 5.7-fold higher than that of the synthetic TNF-α inhibitor SPD304. Molecular docking was performed to determine the binding mode of sennoside B to TNF-α. In conclusion, we successfully developed a novel competition binding screening method to discover small molecule TNF-α inhibitors and identified the natural compound sennoside B as having exceptional potency.

## 1. Introduction

Tumor necrosis factor-alpha (TNF-α), an important pleiotropic cytokine, is a well-known central biological mediator of critical pro-inflammatory and immunomodulatory effects [1,2]. Overexpression of TNF-α is widely associated with a series of autoimmune diseases, including rheumatoid arthritis [3], Crohn’s disease [4], and psoriasis [5]. Because of its profound implications in various diseases, TNF-α has become one of the most promising molecular targets of interest and some therapeutic biologics, such as etanercept, infliximab, certolizumab, golimumab, and adalimumab, have been successfully developed to block the interaction between TNF-α and its receptors [1]. Nevertheless, biological drugs have inherent limitations pertaining to applications because of their immunogenicity, health economics, complexity in manufacturing, and inconvenient routes of administration [1,6,7]. In light of this, a small-molecule drug will have better applicability and be beneficial to more patients.

Suramin was the first small-molecule to demonstrate inhibitory effect on TNF-α through a direct action on the ligand rather than on its receptors [8]. Later, SPD304 was discovered as a small-molecule antagonist of TNF-α with an IC_50_ value of 4.6 µM. X-ray crystallography revealed that SPD304 displaces a subunit of the TNF-α trimer to form a complex with the resultant dimer [9]. Several small-molecule inhibitors that bind to TNF-α or tumor necrosis factor receptor (TNFR) have subsequently been reported. Utilizing a ligand docking-based virtual screening method, Chan et al. discovered two compounds that show a similar binding modality to SPD304, quinuclidine and indoloquinolizidine [10]. Ma et al. applied computer-aided drug design combined with in vitro assays and identified C87 that directly binds to TNF-α [11]. Luzi et al. developed and improved the bicyclic peptide display method and discovered macrocyclic peptide M21, which could dissociate the active trimeric form of TNF-α [12]. Cao et al. used a novel surface plasmon resonance (SPR)-based screening method and identified TNFR1 antagonist physcion-8-O-β-D-monoglucoside (PMG) from complex herbal extracts [13]. Using TNF-α and TNFR crystal structures, Chen et al. performed a virtual screening method followed by SPR and biological validation and discovered T1 and R1, which could bind to TNF-α and TNFR1, respectively [14]. Despite several efforts, compounds’ low potency and potential toxicity impeded further developments and no small molecule drug for TNF-α is currently available [15,16]. Therefore, the discovery of small-molecule TNF-α inhibitors with high efficacy, low toxicity, and potential clinical applications remains a challenging goal.

Natural products (NPs) have played a significant role in the drug discovery and development. According to a recent review paper, among 1394 small-molecule drugs approved over the last four decades, NPs and NP derivatives account for 441 drugs (32%) [17]. The contribution of NPs would be more significant when NP-inspired drugs, such as synthetic drugs with NP pharmacophores or those mimicking NPs, are included in NP drug classification. The virtues of chemical scaffold diversity, structural complexity, and potentially lower toxicity profiles of NPs continuously offers a great opportunity to discover novel bioactive compounds and contribute to drug development.

Encouraged by the fact that natural compounds with high potency and low toxicity that directly target TNF-α have rarely been reported, we developed a competitive binding screening assay coupled with analytical size exclusion chromatography (SEC) and liquid chromatography (LC) tandem mass spectrometry (MS) and applied the screening method to an in-house NP compound library to discover of novel small-molecule TNF-α antagonists. In vitro cell-based assays were performed to validate the inhibitory effect of the NPs and in silico modeling was performed to predict their interactions. Collectively, we observed that sennoside B, a NP small-molecule, effectively inhibited TNF-α and its downstream signaling pathways. Moreover, the binding mode of sennoside B with the TNF-α crystal structure was analyzed using molecular docking, and the key residue interactions between sennoside B and TNF-α were predicted.

## 2. Materials and Methods

### 2.1. Chemicals, Reagents, and Cell Lines

All HPLC grade solvents were purchased from Fisher Scientific (Thermo Fisher Scientific, Hanover Park, IL, USA). Centrifugal ultrafiltration filters (Microcon YM10) were obtained from Millipore (MilliporeSigma, Burlington, MA, USA). Human recombinant TNF-α was obtained from PeproTech (PeproTech, Rocky Hill, NJ, USA). Fetal bovine serum (FBS) was purchased from the American Type Culture Collection (ATCC, Manassas, VA, USA). Prostaglandin D_2_ (PGD_2_) and prostaglandin E_2_ (PGE_2_), and internal standards (IS) of d_4_-prostaglandin D_2_ (d_4_-PGD_2_) and d_4_-prostaglandin E_2_ (d_4_-PGE_2_) were purchased from Cayman Chemical (Cayman Chemical, Ann Arbor, MI, USA). Dimethyl sulfoxide (DMSO), actinomycin D (AMD), and SPD304 were purchased from Sigma-Aldrich (MilliporeSigma, Burlington, MA, USA). L929 and HeLa cells were purchased from ATCC and maintained in Dulbecco’s Modified Eagle Medium (DMEM) supplemented with 10% FBS.

### 2.2. Method Development

#### 2.2.1. Competitive Binding Screening Assay Using Analytical SEC

To develop a competitive binding screening assay, the known TNF-α inhibitor SPD304 and the NP compound were incubated with TNF-α in 70 µL buffer (pH 7.5) consisting of 100 mM Tris, 10% glycerol, 50 mM KCl, and 1 mM EDTA at room temperature for 20 min. The final concentration of SPD304 and the NP compound was 3.5 μM each, and of TNF-α was 0.1 μM. After incubation, the mixture solution was loaded into an SP-6 Bio-gel SEC column (Bio-Rad, Hercules, CA, USA), followed by spin-down at 1000 g for 4 min at 4 °C. The eluted solution containing the ligand and TNF-α complex was washed three times with 50 mM ammonium acetate (pH 7.5), followed by another centrifugation at 13,000 g for 10 min. Then, the ligands were dissociated using 400 μL of methanol. The ultrafiltrate containing the ligand was dried using a SpeedVac (Thermo Fisher Scientific, Hanover Park, IL, USA). The sample was reconstituted with 100 µL of 50% methanol, and 2 µL of the sample was injected into the LC–tandem MS system.

#### 2.2.2. LC–Tandem MS Analysis

The samples were analyzed using an Agilent 1290 HPLC system (Agilent Technologies, Santa Clara, CA, USA) interfaced with a triple quadrupole mass spectrometer (SCIEX API 4000, Foster City, CA, USA) with multiple reaction monitoring (MRM) scan in electrospray ionization positive mode. The separation was achieved using a Waters ACQUITY BEH (Waters Corporation, Milford, MA, USA) C18 column (2.1 × 100 mm, 1.7 µm) fitted to a Waters ACQUITY UHPLC BEH C18 Vanguard pre-column (2.1 × 5 mm, 1.7 µm). The mobile phase consisted of mixtures A (95% water and 5% acetonitrile, 0.1% formic acid) and B (95% acetonitrile and 5% water, 0.1% formic acid). The flow rate was set to 0.6 mL/min. The following gradient program was employed: 0–10 min, 5–100% B. Re-equilibration was applied for 3 min between analyses. The oven temperature was set to 40 °C. The mass spectrometer parameters were as follows: voltage, 5.5 kV; probe temperature, 400 °C; gas 1, 35; gas 2, 40; curtain gas, 40; and dwell time, 200 ms. The MRM parameters for the SPD304 were set up as follows: Q1 (548.4), Q3 (274.3), DP (100), EP (10), CE (43), and CXP (15).

### 2.3. Cell Viability Assay

A cell viability assay was performed using Cell Counting Kit-8 (CCK-8, Dojindo, Kumamoto, Japan) according to the manufacturer’s instruction. Briefly, mouse L929 and human HeLa cells were seeded in 96-well plates at a density of 2.0 × 10^4^ cells/well and cultured overnight. Prepared DMEM containing different concentrations of sennoside B was added to the cells and incubated for 18 h. Subsequently, cells were washed with phosphate-buffered saline three times and cell viability was assessed by adding CCK-8 solution to the treated cells and incubating for 2 h. The optical density in the CCK-8 assay was measured at 450 nm using a microplate reader (BioTek, Winooski, VT, USA).

### 2.4. TNF-α Dependent L929 Cytotoxicity Assay

Treating mouse L929 cells with TNF-α induces cytotoxicity, and the susceptibility of L929 to TNF-α is greatly increased when cells are exposed to AMD [18,19]. This assay has been successfully used to characterize the in vitro efficacy of TNF-α biologics [11,20]. L929 cells were seeded in 96-well plates at a density of 2.0 × 10^4^ cells/well and cultured overnight. Prepared DMEM containing different concentrations of sennoside B (6.25–100 µM), 10 ng/mL of TNF-α, and 1 µg/mL of AMD was added to the cells, and incubated for 18 h. The cell viability was assessed by microscopic examination and CCK-8 assay. SPD304 was used as a positive control.

### 2.5. Western Blot Analysis

L929 and HeLa cell lines were used for western blotting analyses. After the treatment, the cells were lysed and the total protein concentration was determined using Bradford reagent (Bio-Rad, Hercules, CA, USA). Total protein samples (20 µg) were separated using sodium dodecyl polyacrylamide gel electrophoresis and transferred to polyvinylidene difluoride (MilliporeSigma, Burlington, MA, USA) membranes. The membranes were blocked with 5% BSA in TBST and the protein levels of caspase 3, cleaved caspase 3, inhibitor of kappa B-alpha (IκB-α), and glyceraldehyde-3-phosphate dehydrogenase (GAPDH) were determined using anti-caspase 3 primary antibody (#9662, Cell Signaling Technology), anti-cleaved caspase 3 primary antibody (#9661, Cell Signaling Technology), anti-IκB-α primary antibody (#4812, Cell Signaling Technology), and anti-GAPDH primary antibody (#2118, Cell Signaling Technology), respectively. Densitometric analysis of the bands was performed using the LAS4000 system (Fujifilm, Tokyo, Japan).

### 2.6. Measurement of PGD_2_ and PGE_2_ by LC-high Resolution MS

L929 cells were seeded in 6-well plates at a density of 5.0 × 10^5^ cells/well and cultured overnight. Prepared DMEM containing different concentrations of sennoside B (25–100 µM), 10 ng/mL TNF-α, and 1 µg/mL AMD was added to the cells and incubated for 18 h. The collected cell medium was centrifuged at 13,000 g at 4 °C to remove dead cells. Then, 2 µL of d_4_-PGE_2_ and d_4_-PGD_2_ (10 µg/mL, IS) were added to the collected supernatant. The supernatant was cleaned using solid-phase extraction cartridges (Phenomenex, Torrance, CA, USA). The filtrate was dried under nitrogen flow and reconstituted with 100 µL of 50% aqueous methanol, and 2 µL of the reconstituted filtrate was injected directly into the LC-high resolution MS system.

Analysis of PGD_2_ and PGE_2_ was performed according to a previous report [21] using a Vanquish pump and Q-Exactive Hybrid Quadrupole-Orbitrap Mass Spectrometer (Thermo Fischer Scientific, Bremen, Germany). Briefly, the separation was achieved using a Waters ACQUITY BEH (Waters Corporation, Milford, MA, USA) C18 column (2.1 × 100 mm, 1.7 µm) fitted to a Waters ACQUITY UHPLC BEH C18 Vanguard pre-column (2.1 × 5 mm, 1.7 µm). The mobile phase consisted of mixtures A (95% water and 5% acetonitrile, 0.1% formic acid) and B (95% acetonitrile and 5% water, 0.1% formic acid). The flow rate was set to 0.5 mL/min. The following gradient program was employed: 0–10 min, 5–100% B. Re-equilibration was applied for 3 min between analyses. The oven temperature was maintained at 35 °C. The Orbitrap Mass Spectrometer was operated in full scan mode (*m/z* 200–500) in negative ion mode at a mass resolving power of 70,000 (FWHM at *m/z* 200). The electrospray ion source parameters were as follows: spray voltage, 3.5 kV; sheath gas (N_2_ > 95%), 40; auxiliary gas (N_2_ > 95%), 10; sweep gas (N_2_ > 95%), 1; capillary temperature, 320 °C; heater temperature, 300 °C; and S-lens RF level, 50. The full scan mass spectra were acquired using the following parameters: automatic gain control (AGC), 5 × 10^5^ and maximum accumulation time, 100 ms.

### 2.7. Molecular Docking and Dynamic Simulations

The crystal structure TNF-α was retrieved from the Protein Data Bank (ID: 2AZ5). TNF-α and its dimeric form were used as the receptors and sennoside B as the ligand. Molecular docking was performed using the AutoDock 4 [22]. Proteins were prepared by removing water molecules, and the number of rotatable bonds in the ligand was left unmodified. The grid box was set to perform blind docking to cover almost the entire protein structure with a grid spacing of 0.375 Å. Ten docking runs were set with the genetic algorithm, and the maximum number of energy evaluations was changed to 25,000,000 using the Lamarckian genetic algorithm to score the energy. The docking complex of sennoside B with the positive control SPD304 crystal complex structure was scored against TNF-α using the DSX online server [23] to compare interaction scores. Molecular docking interactions and figures were generated using BIOVIA Discovery Studio 2018. GROMACS -2018 [24] was used for molecular dynamics (MD) simulations. The PRODRG server [25] was used to determine the GROMOSA1 force field for the ligand molecules. The proteins and the ligands were solvated with the SPC water model, periodic boundary conditions were applied in all directions, and the total charge of the system was neutralized. Energy minimization steps were carried out using the steepest descent algorithm. For long-range interactions, the particle mesh Ewald method was used with a Fourier spacing of 0.16 nm. The electrostatic cutoff was set to 1.4 nm, and the van der Waals cutoff was set to 1.4 nm. The bond angles were restrained using the LINCS algorithm. The Parrinello–Rahman method was used to set the pressure (1 atm) of the system, and the V-rescale weak coupling method was used to regulate the temperature (310 K). The position restraints in the MD simulations for the NVT and NPT were carried out for 100 ps, with a production run of 30 ns for each protein–ligand complex, and a time step of 2 fs. The structural coordinates were saved every 1 ps, and final snapshots of the complexes were extracted using the GROMACS analysis tool.

### 2.8. Statistical Analysis

All results are presented as the mean ± standard error of the mean (SEM). Statistical analysis was performed using GraphPad Prism 7.0 software (GraphPad Software Inc., San Diego, CA, USA). Statistical significance was considered at *p* < 0.05, after one-way analysis of variance (ANOVA) with Tukey multiple comparison test.

## 3. Results

### 3.1. Development of Competitive Binding Screening Assay

**Figure 1:**
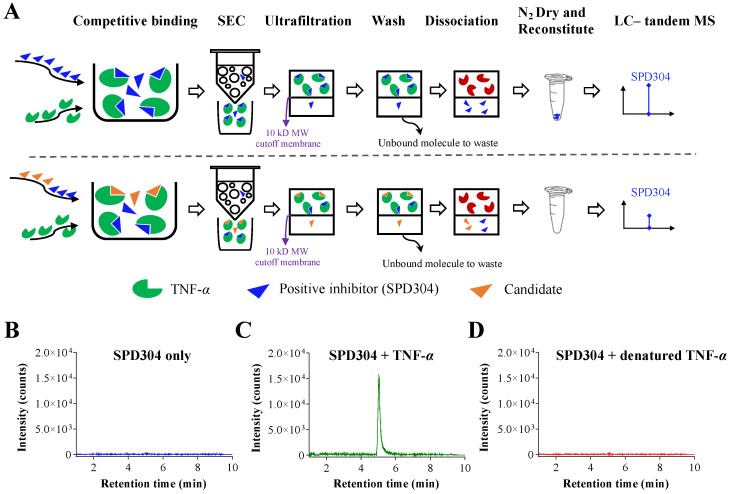
Method development and validation of competitive binding screening assay. (**A**) Schematic overview of competitive binding screening assay. After sample incubation for competitive binding, samples were processed through size exclusion chromatography, washed, and dissociated. The final eluent was N_2_ dried and reconstituted. Samples were analyzed with liquid chromatography-tandem mass spectrometry. The signal intensity of SPD304 when (**B**) SPD304 was incubated without TNF-α; (**C**) SPD304 was incubated with fresh TNF-α; (**D**) SPD304 was incubated with denatured TNF-α.

The scheme illustrating the overall screening method described in the Materials and Methods section is shown in Figure 1A. The known TNF-α inhibitor SPD304 was selected as a positive control or competitive ligand to validate the competitive binding screening assay using analytical SEC LC–tandem MS. First, sample of SPD304 incubated without TNF-α was analyzed as shown in Figure 1B, indicating that the small molecule SPD304 was efficiently removed after SEC and 10 kDa ultrafiltration. Next, when SPD304 was incubated with fresh TNF-α, a strong signal of SPD304 was detected at a retention time of 5.0 min after the process of analytical SEC (Figure 1C). However, no SPD304 signal was detected when SPD304 was incubated with denatured TNF-α (Figure 1D). This result confirmed that signal detection of SPD304 occurred only when it successfully bound to intact TNF-α.

### 3.2. Natural Product Compound Library Screening

**Figure 2:**
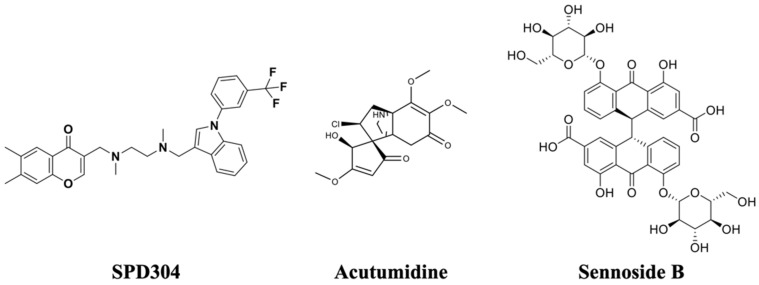
The chemical structures of SPD304, acutumidine, and sennoside B.

Using the validated method, a competitive binding screening assay was performed with the natural product compound library listed in Table 1. Thirty-five prescreened candidate compounds from the library were chemically diverse and included alkaloids, phenolic acids, and flavonoids. These compounds presented at least one of the following bioactivities: anti-inflammatory, anticancer, and antiviral activities. Using SPD304 as a positive control, the competitive binding ability of the library compounds was calculated, and the results are presented in Table 1. Signal reduction of SPD304 suggested competitive binding of a screened compound to TNF-α. In general screening assays, the threshold activity should be determined by making a few considerations, such as test concentration, desired potency, and system sensitivity. In this study, compounds with an inhibition level of SPD304 signal higher than 70% were set as the moderate inhibitors, giving a 5.7% hit rate with two primary hits among the 35 phytochemicals (Table 1). The two hits, sennoside B (#23) and acutumidine (#27), were found to be the most potent inhibitors with inhibition percentages of SPD304 signal at 89.4% and 71.4%, respectively (structures shown in Figure 2).

**Figure 3:**
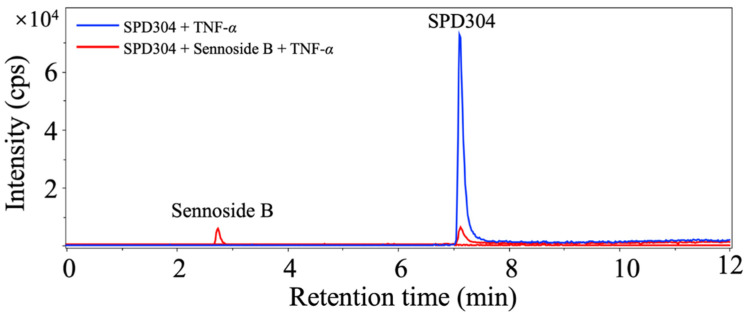
Confirmation of competitive binding of sennoside B with SPD304 against TNF-α. After the competitive binding assay and the subsequent follow-up steps, the samples were analyzed using UPLC-Q Exactive hybrid quadrupole-orbitrap MS. Blue line indicates the signal intensity from SPD304 + TNF-α sample. Red line indicates the signal intensity from SPD304 + sennoside B + TNF-α sample.

To further validate whether the two hits, acutumidine and sennoside B, competitively bind to TNF-α with SPD304, a competition binding assay with the two compounds was performed and the final eluate was analyzed using UPLC-Q Exactive hybrid quadrupole-orbitrap MS. As shown in Figure 3, when SPD304 was incubated with TNF-α alone, the signal of SPD304 was approximately 7 × 10^4^ at a retention time of 7.2 min. However, when sennoside B was added to the mixture of SPD304 and TNF-α, the signal of SPD304 was significantly attenuated to 0.8 × 10^4^. Additionally, the peak of sennoside B was simultaneously detected at a retention time of 2.8 min. These results showed that sennoside B competes with SPD304 for binding to TNF-α. In the case of acutumidine, the result was not reproducible, which led us to focus on sennoside B for further validation.

### 3.3. Sennoside B Inhibits TNF-α-Induced L929 Cell Death

**Figure 4:**
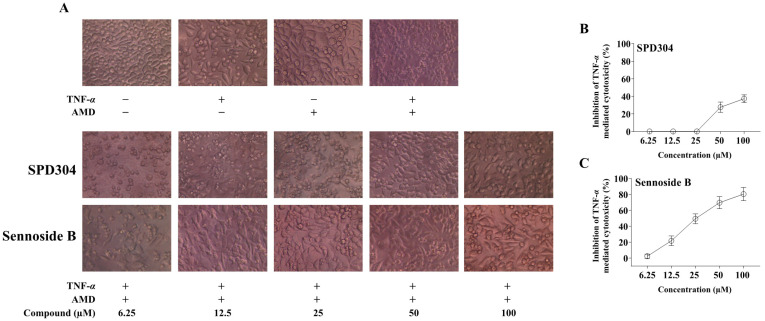
Sennoside B inhibits TNF-α-induced L929 cell death. (**A**) L929 cells were treated with 10 ng/mL TNF-α and 1 µg/mL actinomycin D for 18 h in the presence of the indicated concentrations of SPD304 and sennoside B. SPD304 was used as the positive control. Cell viability was examined under a microscope (×200). Inhibition of TNF-α-mediated cytotoxicity by (**B**) SPD304 and (**C**) sennoside B on L929 cells was measured using the CCK-8 assay. Data were obtained from three independent experiments performed in triplicate and presented as mean ± standard error of the mean (SEM).

Next, we evaluated the efficacy of sennoside B using a TNF-α dependent L929 cell cytotoxicity assay [6,20,26]. In the presence of sub-lethal concentrations of AMD, TNF-α can induce apoptosis in L929 cells. As expected, the positive control SPD304 significantly reduced L929 cell death induced by TNF-α up to 37.33% at 100 µM (Figure 4A,B). Sennoside B showed significant dose-dependent inhibition of TNF-α-mediated cytotoxicity in L929 cells, inhibiting 80.3% at 100 µM concentration (Figure 4A,C). Surprisingly, the efficacy of sennoside B was much better than that of positive control SPD304. When L929 cells were treated with sennoside B alone, a minimal effect on cell proliferation was observed (Supplementary Figure S1), thus excluding the possibility that the TNF-α inhibition efficacy of sennoside B did not result from direct biological effect on L929 cells.

### 3.4. Sennoside B Blocks TNF-α-Induced Signaling Pathways in Mouse L929 Cells

**Figure 5:**
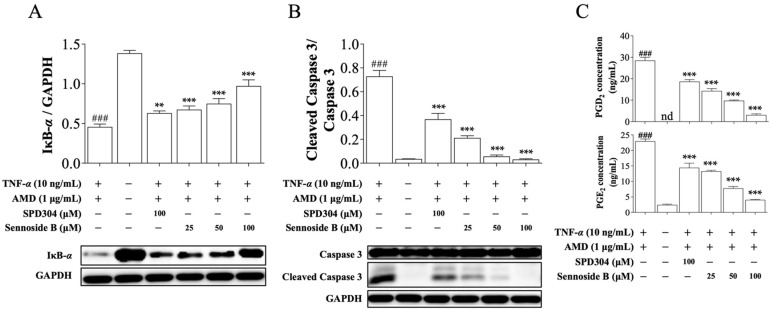
Sennoside B blocks TNF-α-induced signaling pathways in mouse L929 cells. L929 cells were treated with the positive control SPD304 or sennoside B and the protein levels of (**A**) IκB-α, (**B**) caspase 3 and cleaved caspase 3 were measured. GAPDH was used as the internal control. (**C**) After the cell treatment, PGD_2_ and PGE_2_ levels were quantified in the cell medium (*n* = 3). All data are expressed as the mean ± SEM. ** *p* < 0.01 and *** *p* < 0.001 indicate significant difference from the TNF-α-induced group and ^###^ *p* < 0.001 indicates significant difference from the control group. nd indicates not detected.

Having confirmed that sennoside B potently inhibits TNF-α-induced cell death, we next tested the effects of sennoside B on the signaling pathways induced by TNF-α. First, we explored whether sennoside B could block the degradation of IκB-α and the activation of caspase-3, a common downstream signaling process of TNF-α [27,28]. As expected, SPD304 successfully antagonized the downstream signaling pathways of TNF-α in L929 cells (Figure 5). Indeed, sennoside B also significantly inhibited the degradation of IκB-α and the activation of caspase 3 induced by TNF-α in a dose-dependent manner (Figure 5A,B). Eicosanoid lipid mediators, PGD_2_ and PGE_2_, are involved in diverse cellular responses, including cell proliferation, apoptosis, angiogenesis, and inflammation [29]. We found that treatment of L929 cells with TNF-α dramatically increased the secretion of PGD_2_ and PGE_2_ compared with the control group. This effect was significantly inhibited after treatment with 25, 50, and 100 µM of sennoside B (Figure 5C). In line with previous assays, sennoside B showed greater efficacy in blocking the activation of caspase 3 and inhibiting the secretion of pro-inflammatory mediators, compared to SPD304. These data indicate that sennoside B successfully inhibited the inflammatory response induced by TNF-α in mouse L929 cells.

### 3.5. Sennoside B Blocks Degradation of IκB-α in HeLa Cells

**Figure 6:**
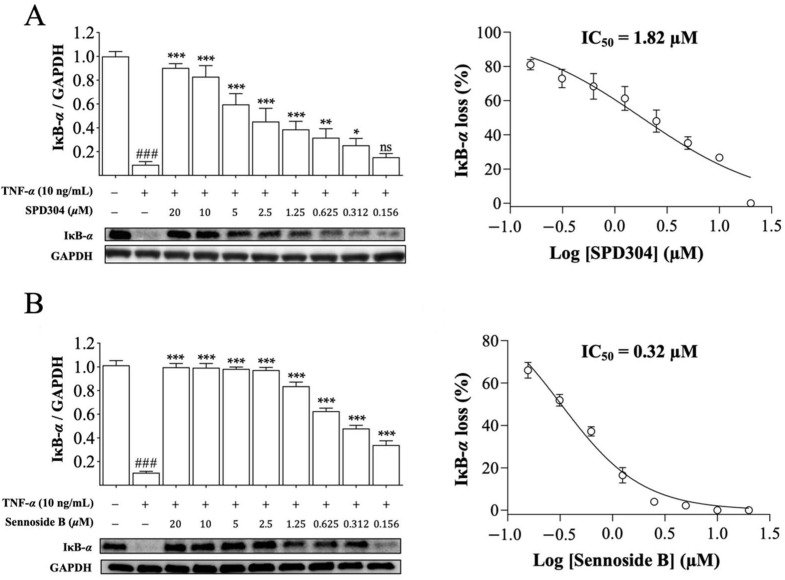
Sennoside B blocks TNF-α induced degradation of IκB-α in human HeLa cells. Cells were treated with the indicated concentrations of (**A**) SPD304 and (**B**) sennoside B in the presence of TNF-α, and the protein level of IκB-α was measured. GAPDH was used as the internal control. The IC_50_ values were calculated based on the inhibition percentage. All data are expressed as the mean ± SEM (*n* = 3). ^###^ *p* < 0.001 indicates significant difference from the control group, and * *p* < 0.05; ** *p* < 0.01, and *** *p* < 0.001 indicate significant difference from the TNF-α-induced group. ns indicates not significant.

After confirming the TNF-α inhibitory effect of sennoside B in a mouse cell line, the inhibitory effect of sennoside B on human cells was investigated. In HeLa cells, SPD304 inhibited TNF-α-mediated IκB-α degradation, and showed a comparable IC_50_ value (1.82 µM), as observed earlier (Figure 6A) [9,10]. Consistently, sennoside B significantly inhibited the degradation of IκB-α induced by TNF-α in HeLa cells and was found to be much more potent than SPD304 with an IC_50_ value of 0.32 µM (Figure 6B), which is 5.69 times lower than that of SPD304. No signs of cytotoxicity or cell proliferation were observed when HeLa cells were treated with sennoside B alone (Supplementary Figure S2). These results suggest that sennoside B can inhibit TNF-α-induced IκB-α degradation in human HeLa cells very well.

### 3.6. Sennoside B-TNF-α Binding Interactions and Stability

**Figure 7:**
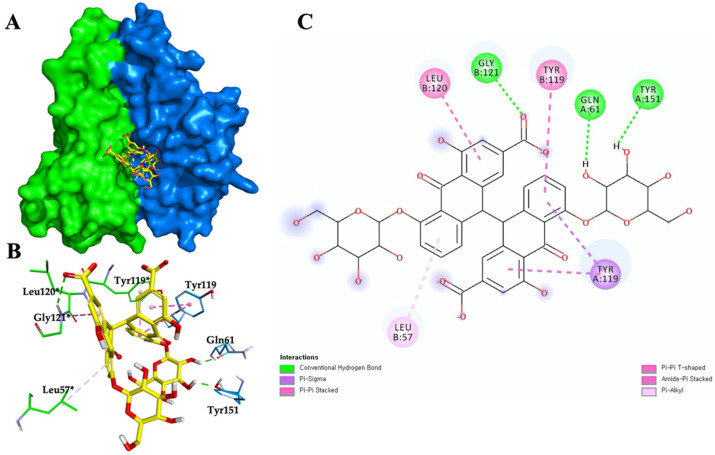
Sennoside B/TNF-α binding interactions and stability. (**A**) Sennoside B binding to the crystal structure of TNF-α (2AZ5). Blue color indicates chain A and green color indicates chain B. (**B**) Three-dimensional representation of sennoside B/TNF-α interactions. Residues of TNF-α and sennoside B are represented as sticks. The carbon and oxygen of ligands are presented in yellow and red colors, respectively. Hydrogen bond and hydrophobic interactions are shown as dotted lines. Asterisks indicate the B chain residues. (**C**) Two-dimensional representation of sennoside B/TNF-α interactions. Carbons of sennoside B are presented in black color.

The binding interactions of SDP304 with the TNF-α dimer include L57, Y59, S60, Q61, Y119, L120, G121, G122, and Y151 from chain A and L57*, Y59*, S60*, Y119*, L120*, G121*, and Y151* from chain B of the dimer (asterisks indicate the TNF-α B chain residues) [9]. Using the TNF-α dimer PDB crystal structure (PDB code 2AZ5), molecular docking was performed to investigate the binding mode of sennoside B to TNF-α. Sennoside B was stably positioned in the hydrophobic cavity of TNF-α and was predicted to have numerous hydrophobic interactions with residues, such as Y119, L57*, Y119*, and L120* (Figure 7). These interactions are also part of the key interactions between TNF-α and the SPD304 complex. Moreover, the significant residues Q61 and Y151 are proposed to form hydrogen bonds with the hydroxyl oxygen atoms in sennoside B, and G121* is proposed to form a hydrogen bond with a carbonyl oxygen atom in sennoside B (Figure 7C). Furthermore, to evaluate the binding energy between TNF-α and sennoside B, binding energy was calculated using the DSX online server and MD simulations. The interaction scores obtained from the DSX online server were –20.730 and –122.948 for SPD304 and sennoside B, respectively. The total interaction energy values obtained using GROMACS 2018 after 30 ns of MD simulations were –246.8415 kJ/mol for SPD304 and –367.8971 kJ/mol for sennoside B. The consistent lowest binding affinity scores of TNF-α/sennoside B suggest that sennoside B is a promising inhibitor of TNF-α. In short, sennoside B may not only fit well in the SPD304 binding site of the TNF-α dimer, but it also forms a complex that appears to be stable in molecular dynamics simulations.

## 4. Discussion

There are several viable strategies to antagonize TNF-α using small molecules. SPD304 directly binds to the inner pocket of the homotrimer of TNF-α and disassembles the homotrimer, resulting in a dimer that has no signaling activity against TNFR [9]. The natural product-derived compound, PMG directly binds to TNFR1, blocking the interaction between TNF-α and TNFR1 [13]. The synthetic compound UCB-9260 uniquely interacts with the homotrimer of TNF-α and stabilizes the asymmetric form of the trimer, which has no signal transduction activity [6]. Resveratrol has been proposed to inhibit TNF-α activity by modulating NF-κB translocation, the downstream signaling of TNF-α [30].

Here, we developed a screening assay based on analytical SEC LC–tandem MS to identify small molecule inhibitors of TNF-α, that can compete for the active site with the known inhibitor SPD304. In silico molecular docking-based virtual screening method offers a fast and cost-effective way to screen diverse chemical libraries against the target of interests, although it requires rigorous validation steps. In vitro protein evolution methods, such as phage display, bacterial display, or mRNA display are powerful methods to screen peptides or proteins that have strong affinities against the desired target, but their utilization for small-molecule screening is limited. Other in vitro affinity-based assays, such as ELISA or SPR, can be used for small molecule screening, but require specific antibodies against the target or target immobilization. Our analytical SEC LC–tandem MS screening method utilizes an intact TNF-α homotrimer in solution without any chemical derivatization on the surface, which allows a much more straightforward and high-throughput screening. In our study, application of this screening method to in-house prescreened NP library compounds led to the identification of sennoside B, which showed higher efficacy than the known inhibitor SPD304.

Sennoside B, a dianthrone glycoside, is one of the main compounds extracted from *Cassia angustifolia*, which has been widely used as a natural laxative in traditional medicines [31]. Additionally, it has been reported that sennoside B can block platelet-derived growth factor receptor signaling in human osteosarcoma cells [32] and inhibit SARS-CoV-2 main protease activity as well [33]. We discovered that sennoside B is a potent inhibitor of TNF-α with an IC_50_ value of 0.32 µM in human HeLa cells. To date, the most potent NP TNF-α inhibitor was quinuclidine, as reported by Chan et al. [10]. Quinuclidine showed an IC_50_ value of 5 µM in TNF-α-induced cell toxicity assays. Although the assayed platform was different, they used SPD304 as a positive control, and upon comparing the biological efficacy of compounds using SPD304, sennoside B showed an IC_50_ value approximately 5.7 times lower than SPD304, whereas quinuclidine had an IC_50_ value 1.6 times higher than SPD304. To our knowledge, sennoside B is by far the most potent NP TNF-α inhibitor known to date. The potency of sennoside B is comparable to that of UCB-9060, the most potent synthetic compound TNF-α inhibitor, with an IC_50_ value of 116 nM in the L929 cell-based assay [6].

Sennoside B is believed to directly bind to the active site of TNF-α, as evidenced by LC-MS detection of both SPD304 and sennoside B. When sennoside B was added to the incubation mixture consisting of SPD304 and TNF-α, and the analytical SEC assay was further processed, the signal of sennoside B appeared, whereas the signal of the positive compound SPD304 was attenuated due to the competitive binding of sennoside B with SPD304 against TNF-α. Molecular docking using the crystal structure (PDB code 2AZ5) of the TNF-α dimer also indicated the possible hydrophobic interactions and hydrogen bonds between sennoside B and the binding site of TNF-α, suggesting that sennoside B effectively binds to and inhibits TNF-α, as specifically described in the results of Figure 7.

## 5. Conclusions

In this study, we developed and validated a competition binding screening method based on analytical SEC coupled with LC–tandem mass spectrometry for the discovery of small-molecule TNF-α inhibitors. Among the screened natural product library compounds, sennoside B showed an exceptional effect on TNF-α inhibition and is probably the most potent natural product TNF-α inhibitor identified to date. The activity of sennoside B was further validated by a series of in vitro cell assays that showed that sennoside B successfully inhibited TNF-α induced cell death and subsequent downstream signaling pathways in both mouse and human cell lines. Finally, the binding mode of sennoside B with the TNF-α crystal structure was analyzed using AutoDock 4, and the key residue interactions between sennoside B and TNF-α were predicted. Currently, the optimization of sennoside B to enhance its pharmacokinetic and metabolic properties is in progress for drug development.

## Figures and Tables

**Table 1:** Natural product compound library list used in the screening and corresponding signal inhibition percentages.

Sample #	Signal Inhibition ^a^	Name	Sample #	Signal Inhibition ^a^	Name
1	18.7	(23)-Hydroxyursolic acid	19	1.3	Gomisin N
2	29.3	Fraxinellone	20	−1.3	Harpagoside
3	20.2	Limonin	21	21.1	(+)-Matrine
4	33.3	Obacunone	22	32.5	Paeoniflorin
5	14.7	Heraclenol	23	89.4	Sennoside B
6	44	Imperatorin	24	25	Tanshinone IIA
7	25.9	Dioscin	25	22.6	Hexahydrocurcumin
8	20	Amygdalin	26	21	Acutumine
9	14.7	Betaine	27	71.4	Acutumidine
10	17.3	Decursin	28	35	Isoliquiritigenin
11	6.9	6,7-Dimethylesculetin	29	12.5	Liquiritin
12	−1.3	Ephedrine	30	17.4	(8)-Demethoxyrunanine
13	15	Eugenol	31	32.5	Chlorogenic acid
14	20	Evodiamine	32	31.2	Caffeine
15	28	(6)-Gingerol	33	0	Dihydrofolic acid
16	33.9	Ginsenoside Rb1	34	23.5	N-(1-Naphthyl)ethylenediamine dihydrochloride
17	17.4	Ginsenoside Rg1	35	15.2	Myricetin
18	25.3	Gomisin A			

^a^: Inhibition percentage (%) of SPD304 signal intensity; # denotes “number”.

## Data Availability

Not applicable.

## Supplementary Materials

The following are available online at https://www.mdpi.com/article/10.3390/biomedicines9091250/s1. Figure S1: Effects of sennoside B on the cell viability of mouse L929 cells. L929 cells were seeded in 96-well plates at a density of 2.0 × 104 cells/well and cultured overnight. Prepared DMEM containing different concentrations of sennoside B (1.56–100 µM) was treated to the cells. After incubating for 18 h, cell viability was measured using the CCK-8 assay, Figure S2: Effects of sennoside B on the cell viability of human HeLa cells. HeLa cells were seeded in 96-well plates at a density of 2.0 × 104 cells/well and cultured overnight. Prepared DMEM containing different concentrations of sennoside B (0.62-40 µM) was treated to the cells. After incubating for 18 h, cell viability was measured using the CCK-8 assay.

## References

[1] Kalliolias G.D., Ivashkiv L.B. (2016). TNF biology, pathogenic mechanisms and emerging therapeutic strategies. Nat. Rev. Rheumatol..

[2] Tracey D., Klareskog L., Sasso E.H., Salfeld J.G., Tak P.P. (2008). Tumor necrosis factor antagonist mechanisms of action: A comprehensive review. Pharmacol. Ther..

[3] Taylor P.C., Feldmann M. (2009). Anti-TNF biologic agents: Still the therapy of choice for rheumatoid arthritis. Nat. Rev. Rheumatol..

[4] Levin A.D., Wildenberg M.E., van den Brink G.R. (2016). Mechanism of Action of Anti-TNF Therapy in Inflammatory Bowel Disease. J. Crohns. Colitis..

[5] Lowes M.A., Bowcock A.M., Krueger J.G. (2007). Pathogenesis and therapy of psoriasis. Nature.

[6] O’Connell J., Porter J., Kroeplien B., Norman T., Rapecki S., Davis R., McMillan D., Arakaki T., Burgin A., Fox Iii D. (2019). Small molecules that inhibit TNF signalling by stabilising an asymmetric form of the trimer. Nat. Commun..

[7] Rider P., Carmi Y., Cohen I. (2016). Biologics for Targeting Inflammatory Cytokines, Clinical Uses, and Limitations. Int. J. Cell Biol..

[8] Alzani R., Cozzi E., Corti A., Temponi M., Trizio D., Gigli M., Rizzo V. (1995). Mechanism of suramin-induced deoligomerization of tumor necrosis factor alpha. Biochemistry.

[9] He M.M., Smith A.S., Oslob J.D., Flanagan W.M., Braisted A.C., Whitty A., Cancilla M.T., Wang J., Lugovskoy A.A., Yoburn J.C. (2005). Small-molecule inhibition of TNF-alpha. Science.

[10] Chan D.S., Lee H.M., Yang F., Che C.M., Wong C.C., Abagyan R., Leung C.H., Ma D.L. (2010). Structure-based discovery of natural-product-like TNF-alpha inhibitors. Angew. Chem. Int. Ed. Engl.

[11] Ma L., Gong H., Zhu H., Ji Q., Su P., Liu P., Cao S., Yao J., Jiang L., Han M. (2014). A novel small-molecule tumor necrosis factor alpha inhibitor attenuates inflammation in a hepatitis mouse model. J. Biol. Chem..

[12] Luzi S., Kondo Y., Bernard E., Stadler L.K., Vaysburd M., Winter G., Holliger P. (2015). Subunit disassembly and inhibition of TNFalpha by a semi-synthetic bicyclic peptide. Protein Eng. Des. Sel..

[13] Cao Y., Li Y.H., Lv D.Y., Chen X.F., Chen L.D., Zhu Z.Y., Chai Y.F., Zhang J.P. (2016). Identification of a ligand for tumor necrosis factor receptor from Chinese herbs by combination of surface plasmon resonance biosensor and UPLC-MS. Anal. Bioanal. Chem..

[14] Chen S., Feng Z., Wang Y., Ma S., Hu Z., Yang P., Chai Y., Xie X. (2017). Discovery of Novel Ligands for TNF-alpha and TNF Receptor-1 through Structure-Based Virtual Screening and Biological Assay. J. Chem. Inf. Model..

[15] McGeary R.P., Bennett A.J., Tran Q.B., Cosgrove K.L., Ross B.P. (2008). Suramin: Clinical uses and structure-activity relationships. Mini. Rev. Med. Chem..

[16] Sun H., Yost G.S. (2008). Metabolic activation of a novel 3-substituted indole-containing TNF-alpha inhibitor: Dehydrogenation and inactivation of CYP3A4. Chem. Res. Toxicol..

[17] Newman D.J., Cragg G.M. (2020). Natural Products as Sources of New Drugs over the Nearly Four Decades from 01/1981 to 09/2019. J. Nat. Prod..

[18] Rubin B.Y., Smith L.J., Hellermann G.R., Lunn R.M., Richardson N.K., Anderson S.L. (1988). Correlation between the anticellular and DNA fragmenting activities of tumor necrosis factor. Cancer Res..

[19] Trost L.C., Lemasters J.J. (1994). A cytotoxicity assay for tumor necrosis factor employing a multiwell fluorescence scanner. Anal. Biochem..

[20] Nesbitt A., Fossati G., Bergin M., Stephens P., Stephens S., Foulkes R., Brown D., Robinson M., Bourne T. (2007). Mechanism of action of certolizumab pegol (CDP870): In vitro comparison with other anti-tumor necrosis factor alpha agents. Inflamm. Bowel. Dis..

[21] Shin J.S., Peng L., Kang K., Choi Y. (2016). Direct analysis of prostaglandin-E2 and -D2 produced in an inflammatory cell reaction and its application for activity screening and potency evaluation using turbulent flow chromatography liquid chromatography-high resolution mass spectrometry. J. Chromatogr. A.

[22] Morris G.M., Huey R., Lindstrom W., Sanner M.F., Belew R.K., Goodsell D.S., Olson A.J. (2009). AutoDock4 and AutoDockTools4: Automated docking with selective receptor flexibility. J. Comput. Chem..

[23] Gohlke H., Hendlich M., Klebe G. (2000). Knowledge-based scoring function to predict protein-ligand interactions. J. Mol. Biol..

[24] Pronk S., Pall S., Schulz R., Larsson P., Bjelkmar P., Apostolov R., Shirts M.R., Smith J.C., Kasson P.M., van der Spoel D. (2013). GROMACS 4.5: A high-throughput and highly parallel open source molecular simulation toolkit. Bioinformatics.

[25] Schuttelkopf A.W., van Aalten D.M. (2004). PRODRG: A tool for high-throughput crystallography of protein-ligand complexes. Acta Crystallogr. D.

[26] Humphreys D.T., Wilson M.R. (1999). Modes of L929 cell death induced by TNF-alpha and other cytotoxic agents. Cytokine.

[27] Faustman D., Davis M. (2010). TNF receptor 2 pathway: Drug target for autoimmune diseases. Nat. Rev. Drug Discov..

[28] Van Herreweghe F., Festjens N., Declercq W., Vandenabeele P. (2010). Tumor necrosis factor-mediated cell death: To break or to burst, that’s the question. Cell Mol. Life Sci. CMLS.

[29] Nakanishi M., Rosenberg D.W. (2013). Multifaceted roles of PGE2 in inflammation and cancer. Semin. Immunopathol..

[30] Silva A.M., Oliveira M.I., Sette L., Almeida C.R., Oliveira M.J., Barbosa M.A., Santos S.G. (2014). Resveratrol as a natural anti-tumor necrosis factor-alpha molecule: Implications to dendritic cells and their crosstalk with mesenchymal stromal cells. PLoS ONE.

[31] Wu H., Feng F., Jiang X., Hu B., Qiu J., Wang C., Xiang Z. (2020). Pharmacokinetic and metabolic profiling studies of sennoside B by UPLC-MS/MS and UPLC-Q-TOF-MS. J. Pharm. Biomed. Anal..

[32] Chen Y.C., Chang C.N., Hsu H.C., Chiou S.J., Lee L.T., Hseu T.H. (2009). Sennoside B inhibits PDGF receptor signaling and cell proliferation induced by PDGF-BB in human osteosarcoma cells. Life Sci..

[33] Abdallah H.M., El-Halawany A.M., Sirwi A., El-Araby A.M., Mohamed G.A., Ibrahim S.R.M., Koshak A.E., Asfour H.Z., Awan Z.A., Elfaky M.A. (2021). Repurposing of Some Natural Product Isolates as SARS-COV-2 Main Protease Inhibitors via In Vitro Cell Free and Cell-Based Antiviral Assessments and Molecular Modeling Approaches. Pharmaceuticals.

