# Supplementary material for: A Novel Competitive Binding Screening Assay Reveals Sennoside B as a Potent Natural Product Inhibitor of TNF-α
Source: Biomedicines. 2021 Sep 17;9(9):1250. doi: 10.3390/biomedicines9091250 (PMC8465676; doi:10.3390/biomedicines9091250)
Supplement: Supplementary file 1 [file biomedicines-09-01250-s001.zip › biomedicines-1348369-supplementary.pdf]

Supplementary Materials

A Novel competitive binding screening assay reveals sennoside B as a potent natural product inhibitor of TNF-α

Lei Peng[1], Prasannavenkatesh Durai[2], Keunwan Park[2], Jeong Joo Pyo[3,*], and Yongsoo Choi[3,4,*]

1 School of Chemistry and Chemical Engineering, Qiqihar University, Qiqihar, Heilongjiang, China; 03555@qqhru.edu.cn (L.P.)

2 Natural Product Informatics Research Center, Korea Institute of Science and Technology (KIST), Gangneung 25451, Republic of Korea; prasanna@kist.re.kr (P.D.); keunwan@kist.re.kr (K.P.)

3 Natural Product Research Center, Korea Institute of Science and Technology (KIST), Gangneung 25451, Republic of Korea

4 Department of Biological Chemistry, University of Science and Technology, Daejeon 305-350, Republic of Korea

*Correspondence: brianpyo@kist.re.kr (J.J.P.); yongsoo.choi@kist.re.kr (Y.C.)

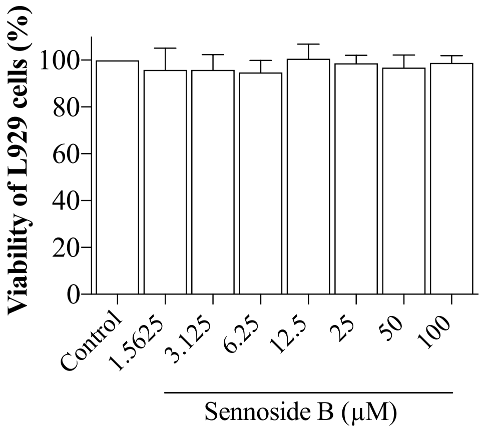

Figure S1. Effects of sennoside B on the cell viability of mouse L929 cells. L929 cells were see ded in 96-well plates at a density of 2.0 × 10^4 cells/well and cultured overnight. Prepared DM EM containing different concentrations of sennoside B (1.56-100 μM) was treated to the cells. After incubating for 18 h, cell viability was measured using the CCK-8 assay.

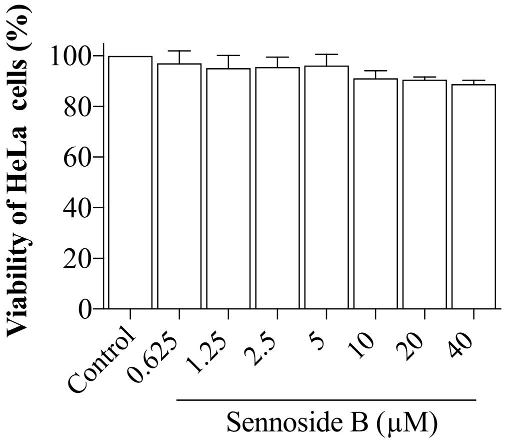

Figure S2. Effects of sennoside B on the cell viability of human HeLa cells. HeLa cells were s eeded in 96-well plates at a density of 2.0 × 10^4 cells/well and cultured overnight. Prepared D MEM containing different concentrations of sennoside B (0.62-40 μM) was treated to the cells. After incubating for 18 h, cell viability was measured using the CCK-8 assay.
